# Does bearing size influence metal ion levels in large-head metal-on-metal total hip arthroplasty? A comparison of three total hip systems

**DOI:** 10.1186/1749-799X-9-3

**Published:** 2014-01-28

**Authors:** James Smith, David Lee, Kamal Bali, Pam Railton, David Kinniburgh, Peter Faris, Deborah Marshall, Brian Burkart, James Powell

**Affiliations:** 1 Arthroplasty Section, Faculty of Medicine, University of Calgary, #0444 3134 Hospital Drive NW, Calgary AB T2N 5A1, Canada; 2 Alberta Bone and Joint Health Institute, 3280 Hospital Drive NW #400, Calgary AB T2N 4Z6, Canada; 3 Alberta Centre for Toxicology, Calgary AB T2N 4 N1, Canada; 4 Alberta Health Services, 10101 Southport Rd. SW, Calgary AB T2W 3 N2, Canada; 5 Orthopaedic Trauma and Lower Extremity Reconstruction, University of Calgary, #0444 3134 Hospital Drive NW Calgary AB T2N 5A1, Canada

## Abstract

**Background:**

The purpose of the study was twofold: first, to determine whether there is a statistically significant difference in the metal ion levels among three different large-head metal-on-metal (MOM) total hip systems. The second objective was to assess whether position of the implanted prostheses, patient demographics or factors such as activity levels influence overall blood metal ion levels and whether there is a difference in the functional outcomes between the systems.

**Methods:**

In a cross-sectional cohort study, three different metal-on-metal total hip systems were assessed: two monoblock heads, the Durom socket (Zimmer, Warsaw, IN, USA) and the Birmingham socket (Smith and Nephew, Memphis, TN, USA), and one modular metal-on-metal total hip system (Pinnacle, Depuy Orthopedics, Warsaw, IN, USA). Fifty-four patients were recruited, with a mean age of 59.7 years and a mean follow-up time of 41 months (12 to 60). Patients were evaluated clinically, radiologically and biochemically. Statistical analysis was performed on all collected data to assess any differences between the three groups in terms of overall blood metal ion levels and also to identify whether there was any other factor within the group demographics and outcomes that could influence the mean levels of Co and Cr.

**Results:**

Although the functional outcome scores were similar in all three groups, the blood metal ion levels in the larger monoblock large heads (Durom, Birmingham sockets) were significantly raised compared with those of the Pinnacle group. In addition, the metal ion levels were not found to have a statistically significant relationship to the anteversion or abduction angles as measured on the radiographs.

**Conclusions:**

When considering a MOM THR, the use of a monoblock large-head system leads to higher elevations in whole blood metal ions and offers no advantage over a smaller head modular system.

## Introduction

Metal on polyethylene (MOP) has traditionally been the most frequently used bearing system in total hip arthroplasty (THA) [1]. However, wear-induced osteolysis continues to be a common cause of failure for this bearing surface [1,2]. Metal-on-metal (MOM) THA has emerged as an attractive alternative bearing surface in THA because of its potential for decreased wear and reduced dislocation rates [3,4].

Although the cobalt-chromium alloys used for MOM THA have decreased volumetric wear [5], they have been shown to release up to 500 times more particles as compared to the MOP implants [6]. These released metal particles can undergo oxidation, reabsorption and eventual release in the blood [7]. The metal ion levels in patients with MOM implants have been shown to be higher as compared to patients with other bearing surfaces [8,9]. The increasing number of joint replacements especially in younger patients who are exposed to orthopaedic metal alloys is raising questions as to the long-term effects of exposure. Concerns include possible local tissue toxicity, hypersensitivity, altered lymphocyte concentrations, osteolysis, irreversible chromosomal damage and carcinogenicity [10-15]. Arthroprosthetic cobaltism is now a recognized serious complication characterized by neurological, endocrine and cardiac symptoms [16-18].

Several clinical and *in vitro* hip simulator studies have shown a number of factors that influence the amount of metal ions produced in MOM bearing surfaces. These have primarily included modularity of the prostheses [19-23], overall position of the implanted components [24-28] and the size of the femoral head [23,28-36]. Most of the clinical studies related to metal ion blood levels in MOM bearing surfaces have evaluated only MOM hip resurfacing (to see the effect of component position) [25-28] or compared large-head MOM hip resurfacing with small-head MOM THA (to see the effect of femoral head size) [23,28-30,32,33,36].

Although the bearing surface is similar in both MOM hip resurfacing and MOM THA, the corrosion at the head-neck junction (trunnion) in MOM THA can be an additional source of metal ion release and this could be dependent on the femoral head size [19-23]. A few studies have actually looked at the effect of femoral head size [31,34,35] on the metal ion levels in MOM THA. However, these studies have mainly compared metal ion levels in small-head (<36 mm) and large-head (≥36 mm) MOM THA. Corrosion at the head-neck junction can be particularly problematic in large diameter MOM THA because of higher friction, which increases rotational movement at the head and neck junction [20]. This in turn can produce high metal ion levels. Whether there could be any differences in metal ion levels due to component size or specific implant type in large-head MOM THA has not yet been thoroughly studied. To our knowledge, there are only two studies [20,37] comparing metal ions and clinical outcomes of different large-head MOM total hip prostheses. However, these studies did not evaluate the effect of femoral head size on metal ion levels in their series of large-head MOM THA.

The primary objective of this study was to evaluate the distribution of metal ion levels among the three different large-head MOM total hip systems: two monoblock large-head systems (the Durom Socket, Zimmer, Warsaw, IN, USA and the Birmingham socket, Smith and Nephew, Memphis, TN, USA) and one modular metal-on-metal total hip system (Pinnacle, Depuy Orthopedics, Warsaw, IN, USA). The secondary objectives were to assess whether position of the implanted prostheses, patient demographics or factors such as activity levels influence overall blood metal ion levels and whether the short-term functional outcome differs with specific large-head MOM implant type.

## Materials and methods

This was a cross-sectional cohort study recruiting 54 patients who had received three different large-head MOM prostheses. The three hip systems included two monoblock large heads (Durom or Birmingham socket) and one modular system (Pinnacle). All the stems used for these implants had a 12/14 taper for head-neck junction. The exclusion criteria included diagnosis other than osteoarthritis, infection, metal components elsewhere in the body and renal insufficiency. The minimum time for follow-up was selected as 1 year to allow for the fact that metal ion levels are known to be elevated in the initial run-in period lasting for approximately 1 year postoperatively [3,35].

The mean age of the patients was 59.7 years with the mean follow-up time of 41 months (range, 12 to 60 months). There were 19 patients in each of the monoblock large head groups (Durom or Birmingham socket) operated by a single surgeon (JP). The third modular Pinnacle group had 16 patients operated by another surgeon (BB). Both the surgeons used a posterior approach to expose the hip and cementless fixation of the components. The median head size used in the Birmingham group was 52 mm (range 44 mm to 56 mm) and in the Durom group, 48 mm (range 42 to 54 mm). The median head size used in the modular Pinnacle group was 40 mm (range 36 to 44 mm).

Following institutional review board approval, all the patients were contacted by telephone and asked if they would be interested in participating in the study. Patients agreeing to participate were invited to attend dedicated clinics for clinical, radiological and biochemical evaluation.

### Clinical evaluation

This included assessment of Harris hip score, Western Ontario and McMaster Universities (WOMAC) index, short form 36 (SF 36), and University of California Los Angeles (UCLA) activity score (1, ‘no physical activity, dependent on others’ to 10, ‘regular participation in impact sports’). Range of motion (ROM) was calculated as part of the Harris hip score.

### Biochemical evaluation

Venous whole blood samples were obtained for cobalt (Co) and chromium (Cr) levels. Blood in the first 40 patients assessed was initially sent to two separate laboratories, Trace elements Laboratory, London, Ontario, Canada and Alberta Centre for Toxicology, Calgary, Alberta. Both the laboratories utilized an octopole reaction system (ORS) inductively coupled plasma mass spectrometer (ICPMS) for measuring the whole blood metal ion levels. The ORS-ICPMS method has been previously described by Pei et al. [38]. Due to a high correlation in metal ion results between the laboratories, the final group of patients was assessed in just one location (Calgary). All blood ion levels from the Calgary laboratory were used in our data analysis.

### Radiological evaluation

Standardized anteroposterior (AP) and cross table lateral radiographs of the pelvis and hip were assessed. All AP radiographs were taken with the legs in 15° of internal rotation. The best pre- and postoperative films were selected for each patient and assessed using Dicom software (United Kingdom) for calculation of inclination and anteversion of the acetabular components.

### Statistical analysis

This was performed on all the collected data to assess for any differences between the three groups in terms of demographics, overall blood metal ion levels, acetabular component position and clinical outcomes. As this was an exploratory study, we did not conduct an *a priori* sample size calculation. Analysis of variance or *t* tests were used for the comparisons of means (for continuous variables with near symmetrical distribution) across device type. For continuous variables that did not have approximately symmetrical and normal distributions, the medians (with the 25th percentile and 75th percentile) were calculated. Mann–Whitney non-parametric tests were used for comparisons of these variables across device types (large modular vs Pinnacle devices). Binary (yes/no) and categorical variables were evaluated as percentages. Corrected *χ*^2^ tests were used to compare binary and categorical variables (calculated as percentages) across device types. We considered two-tailed probability values <0.05 to indicate statistical significance for all statistical tests.

## Results

**Figure 1:**
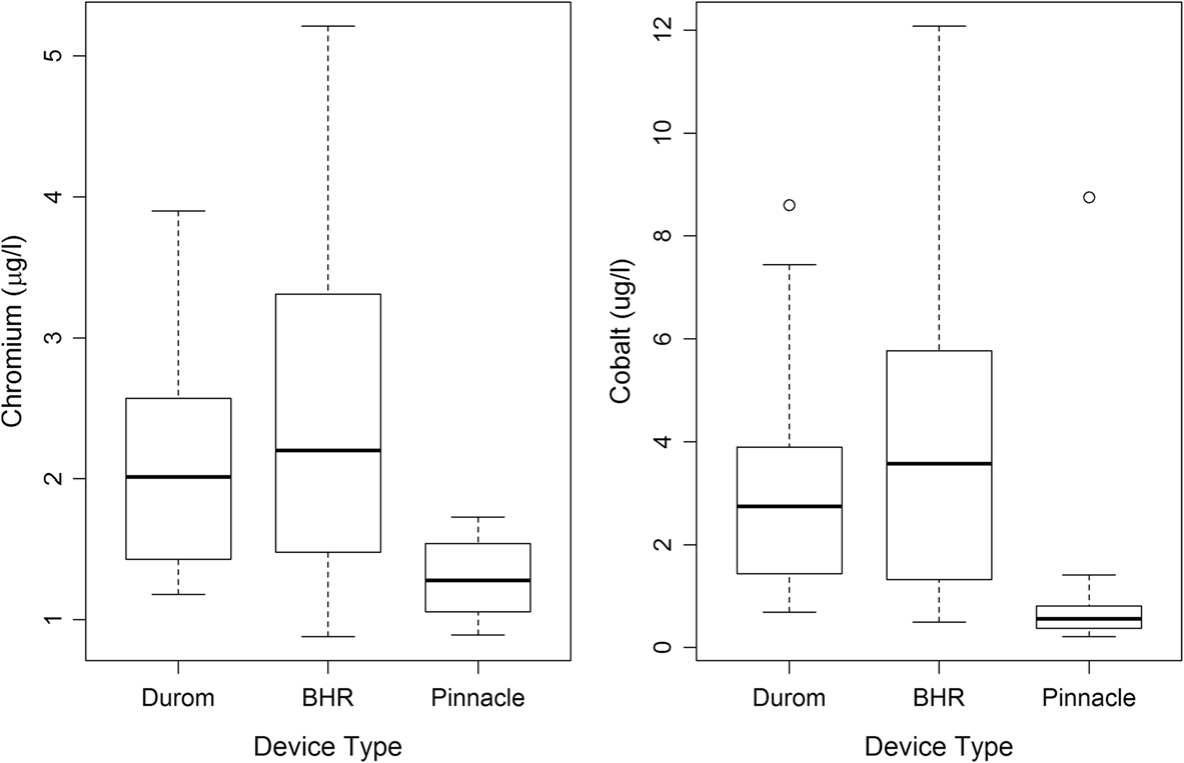
**Box and whisker plots showing the distributions of chromium and cobalt levels for all patients by implant type.** For each device type, the top and bottom of the ‘box’ represents the 75th and 25th percentiles of the data. The heavy horizontal line through the box is the median. Circles outside of the ‘whiskers’ represent outliers. The top and bottom of the whiskers are the maximum and minimum values that are not outliers.

The blood metal ion levels in the larger monoblock large heads (with a Durom or Birmingham socket) were significantly raised compared to the Pinnacle group (Figure 1). Median Co levels were 2.8 and 3.3 μg/l in the Durom and Birmingham groups, respectively, compared to only 0.52 μg/l in the Pinnacle group (*p* < 0.001). Median Cr levels were 2 and 2.2 μg/l in the Durom and Birmingham groups, respectively, compared to only 1.2 μg/l in the Pinnacle group (*p* < 0.001). In all the groups, however, the whole blood metal ion levels were within an acceptable safe range when compared to the upper safe limit of metal ion levels as set forth by various studies [39-42].

**Figure 2:**
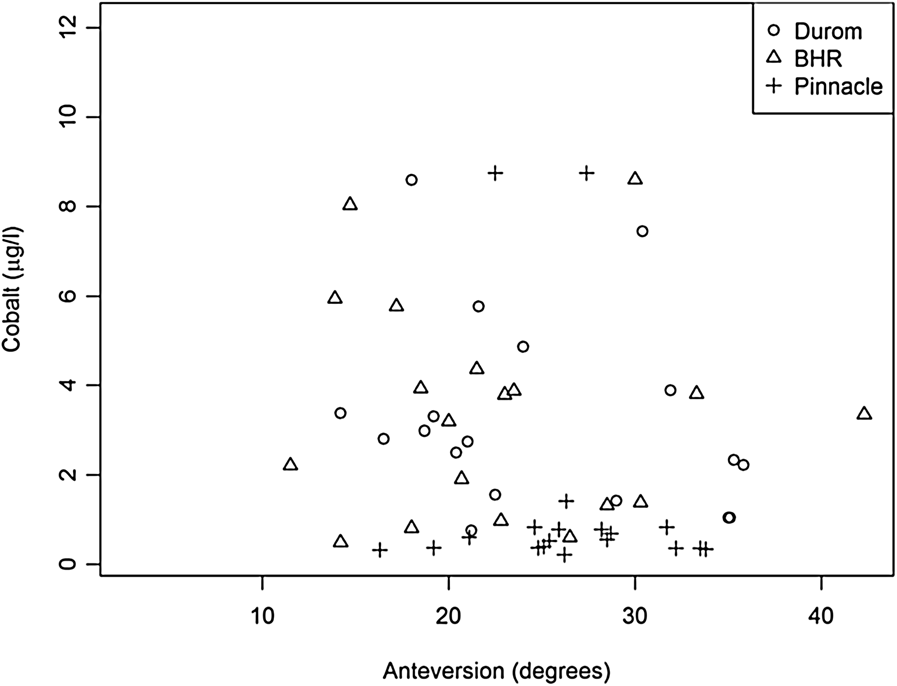
Relationship between acetabular anteversion and cobalt levels by implant type.

**Figure 3:**
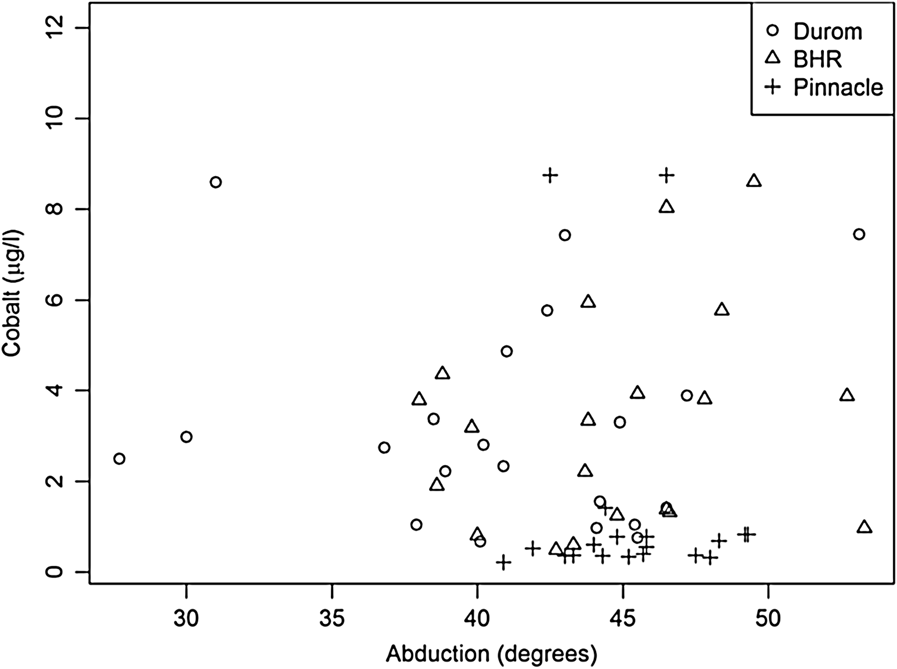
Relationship between acetabular abduction angle and cobalt levels by implant type.

Although there were observed differences in abduction and anteversion angles for the different devices (Figures 2 and 3), these differences were not shown to be statistically significant. Further, the abduction and anteversion angles were found to be within acceptable limits for most of the patients and did not seem to correlate with blood metal ion levels (Figures 2 and 3).

As summarized in Table 1, there were no statistically significant differences between the three groups in terms of age, sex, functional outcome, UCLA activity score or follow-up time. The ROM scores (calculated as part of the Harris Hip Scores) were also not significantly different between the three groups. None of these factors was found to be related to whole blood metal ion levels in the patients. There was no dislocation in any of the patients. The only complication reported was infection following a dental abscess in one of the patients in the Durom group.

**Table 1:** Patient characteristics by implant type

	**Durom**	**Birmingham**	**Pinnacle**	***p *** **value**^**a**^
Age in years (mean ± SD)	61.7 ± 5.9	60.4 ± 11.3	56.5 ± 9.2	0.2251
Female sex	31.6%	73.7%	93.8%	0.0003
	(6/19)	(14/19)	(15/16)	
BMI (mean ± SD)	30.2 ± 7.1	30.7 ± 7.4	28.4 ± 4.3	0.5559
Diabetes	5.3%	5.3%	0.0%	1.0000
	(1/19)	(1/19)	(0/16)	
Smoking	15.8%	21.1%	12.5%	0.9011
	(3/19)	(4/19)	(2/16)	
SF-36 (mean ± SD)	85.6 ± 12.0	84.1 ± 12.5	79.6 ± 18.7	0.4639
Harris hip score (mean ± SD)	80.0 ± 15.9	76.4 ± 15.3	78.1 ± 16.9	0.7832
UCLA activity scale (mean ± SD)	6.5 ± 1.7	7.1 ± 1.7	7.1 ± 1.3	0.4456
WOMAC score (mean ± SD)	84.3 ± 11.8	82.2 ± 13.7	80.7 ± 13.2	0.7122
Cobalt (μg/l) median (25th, 75th percentile)	2.8	3.3	0.5	<0.001
	(1.5,3.6)	(1.4,4.1)	(0.4,0.7)	
Chromium (μg/l) median (25th, 75th percentile)	2.0	2.2	1.2	<0.001
	(1.5,2.5)	(1.5,2.7)	(1.0,1.4)	
Head size (mm) median (25th, 75th percentile)	48.0	48.0	40.0	<0.0001
	(44.0,50.0)	(44.0,54.0)	(40.0,40.0)	

^a^*p* value (Pinnacle vs Durom and Birmingham implants).

## Discussion

Measurement of metal ion levels in the blood following MOM arthroplasty is important as higher levels have been found to be associated with prosthetic failure [21-23,37]. There have also been reports of metal ion toxicity in a few patients [16-18]. Unfortunately with the large numbers of different combinations of implants [29,30,34,39] as well as different methods of analysis to determine minute traces of metal ion levels with the blood, it is difficult to compare published studies on this subject. A safe level of metal ions in blood is also hard to define [40]. A serum Cr level of >17 μg/L and a Co of >19 μg/l is more likely to be associated with metallosis leading to tissue damage [40-42]. The United Kingdom’s Medicines and Healthcare Products Regulatory Agency [42] has advised that patients with MOM implants who have pain, prosthetic malposition or implants that are known to have excessive failure rates should be evaluated with the measurement of serum cobalt. Whilst normal serum cobalt and chromium levels are known and it is recognized that they are elevated following metal-on-metal THR, toxic levels are yet to be established [40]. Every effort should be made to try and reduce these when considering MOM arthroplasty.

The current study was primarily aimed at evaluating blood metal ions in three commonly used larger head MOM THA systems. These hip systems included sockets from Zimmer (Durom), Smith and Nephew (Birmingham) and Depuy (Pinnacle). The Durom is a one-piece metal socket device with plasma coating. The Birmingham cup is also a one-piece metal acetabular socket with beads on the outer surface coated with hydroxyapatite. Both these monoblock sockets articulate with a large metal head. The Pinnacle, on the other hand, is a modular system with a metal liner (Ultamet) sitting inside the metal shell. The size of the metal head that articulates with the metal liner in the Pinnacle system, although larger than traditional metal on poly bearings, is smaller than the head size in the monoblock systems (Durom or Birmingham).

The most important finding of the study was a clear demonstration of statistically significant differences in the metal ion levels in patients following a monoblock large-head MOM arthroplasty system compared to a smaller modular MOM hip arthroplasty. In our series, the smaller head size (36 to 44 mm) appeared to produce less metal ions whilst at the same time was large enough to increase hip stability and provide a range of motion and function comparable to the larger head size (>44 mm).

Size of the femoral head has been shown to be an important predictor of blood metal ion levels in MOM arthroplasty [23,28-36]. Although a larger femoral head size theoretically produces less volumetric wear [3] (and hence lower metal ions in blood), this has not been consistently shown to be the case in various studies on the subject. Most of the studies evaluating the effect of femoral head size on blood metal ion levels have specifically looked for the same in hip resurfacing procedures or compared ion levels in MOM resurfacing and MOM total hip arthroplasty [28-30,33,36,43]. These studies have been summarized in Table 2. However, a MOM THA may behave differently as compared to the MOM resurfacing as far as the amount of metal ions released is concerned. This could be due to a higher modularity of MOM THA systems and the presence of additional MOM surfaces like the head-neck junction, which could be prone to corrosion and metal ion release [19-23]. As such, the effect of femoral head size in a MOM THA may be different from MOM resurfacing. Only four studies, to our knowledge, have compared the metal ion levels in small and large-head MOM THA [31,32,34,35]. However, none of these found metal ion levels to be consistently dependent on the size of the femoral head. These studies are summarized in Table 3.

**Table 2:** Effect of femoral head size on metal ion concentrations

**Authors**	**Year**	**Study design**	**Follow-up**	**Metal ion levels (μg/l)**	***p *** **value**	**Conclusion**
Clarke et al. [29]	2003	Comparison of large-head MOM resurfacing (38 to 54) with 28 mm head MOM THA	16 months	Large head, Cr 53, Co 38	0.0001 for Cr	Metal ion levels higher for larger diameter bearings
				Small head: Cr 29, Co 22 (all values nmol/l)	0.0021 for Co	
Daniel et al. [30]	2006	Comparison of large-head MOM resurfacing (50 to 54) with 28 mm head MOM THA	12 months	Large head, Cr 1.3, Co 2.4	0.055 for Cr	No difference in metal ion levels in small or large-head MOM bearings
				Small head, Cr 1.7, Co 1.7	0.28 for Co	
Langton et al. [28]	2008	Comparison of large-head MOM resurfacing (≥53 mm) with small-head MOM resurfacing (≤53 mm)	26 months	Large head, Cr 3.04, Co 1.48	0.004 for Cr	Metal ion levels higher for smaller diameter MOM resurfacings
				Small head, Cr 4.12, Co 2.43	0.007 for Co	
Vendittoli et al. [33]	2010	Comparison of large-head MOM resurfacing (40 to 58) with 28 mm head MOM THA	24 months	Large head, Cr 1.58, Co 0.67	0.819 for Cr	No difference in metal ion levels in small or large-head MOM bearings
				Small head, Cr 1.62, Co 0.94	0.207 for Co	
Pattyn et al. [36]	2011	Comparison of large-head MOM resurfacing (Durom and BHR) with 28 mm head MOM THA	24 months	Large-head Durom, Cr 1.07, Co 0.79	Significant only in Co levels (Durom vs small head MOM THA)	Metal ions lower in larger head Durom resurfacing as compared to small-head MOM THA. No difference between BHR and small-head MOM THA
				Large-head BHR, Cr 1.54, Co 1.86		
				Small head, Cr 1.25, Co 1.79		
Moroni et al. [42]	2008	Comparison of large head MOM resurfacing (average diameter 48) with 28 mm head MOM THA	24 months	Large head, Cr 2.3, Co 1.4	0.06 for Cr	No difference in metal ion levels in small or large-head MOM bearings
				Small head, Cr 1.73, Co 1.33	0.30 for Co	

Studies evaluating MOM hip resurfacing alone or comparing MOM resurfacing with small-head MOM THA.

**Table 3:** Effect of femoral head size on metal ion concentrations

**Authors**	**Year**	**Study design**	**Follow-up**	**Metal ion levels (μg/l)**	***p *** **value**	**Conclusion**
Antoniou et al. [32]	2008	Comparison of 36 mm head MOM THA with 28 mm head MOM THA	12 months	Large head, Cr 0.4, Co 2.3	>0.2 for Cr	No difference in metal ion levels in small or large-head MOM THA
				Small head, Cr 0.6, Co 2.6	>0.15 for Co	
Daniel et al. [31]	2008	Comparison of large-head MOM THA (42 to 54) with 28 mm head MOM THA	12 months	Large head, Cr 1.4, Co 2.3	Not significant	No difference in metal ion levels in small or large-head MOM THA
				Small head, Cr 1.7, Co 1.7		
Bernstein et al. [34]	2011	Comparison of large-head MOM THA (40 to 44) with small-head MOM (28 and 36 mm) THA	12 months	Large head, Cr 0.51, Co 2.22	0.29 for Cr	No difference in metal ion levels in small or large-head MOM THA
				Small head, Cr 0.78, Co 2.34	0.42 for Co	
Hallows et al. [35]	2011	Comparison of large-head MOM THA (38 and larger) with small-head MOM (28 and 32 mm) THA	12 months	Large head, Cr 0.8, Co 0.7	0.0158 for Cr	Chromium ion levels in blood higher for small-head MOM THA as compared to large-head MOM THA. No difference in blood Cobalt ion levels
				Small head, Cr 2.1, Co 0.7	0.869 for Co	

Studies comparing small-head MOM THA with large-head MOM THA.

There have been several reports over the last few years suggesting a higher than anticipated failure rate of large-head monoblock MOM prostheses. This has raised concern and has led not only to the withdrawal of some devices from the market but also advice from the British Orthopaedic Association to consider carefully and possibly avoid the use of large diameter MOM bearings [44]. These failures are related to higher circulating levels of metal ions in the blood [37].

There are a number of implant design factors that could potentially influence the production of metal ions including the fluid film to surface roughness ratio (Lambda ratio), the surface roughness of a material as well as hardness and sphericity [45]. However, it has not yet been clearly established if specific implant types are related to the higher failure rate of large-head MOM THA and if metal ion levels (which correlate with failure) differ with different large-head MOM THA implant systems. Only two studies [20,37] prior to the current study have actually looked at that. These studies have been summarized in Table 4.

**Table 4:** Comparison of metal ion levels and clinical outcomes in various large-head MOM THA systems

**Authors**	**Study design**	**Metal ion levels**	**Clinical outcomes**
Lavigne et al. [20]	Comparison of metal ion levels in four different large-head MOM THA systems (Biomet modular M2a-Magnum system, Depuy ASR XL system, Smith and Nephew Birmingham socket system and Zimmer Durom LDH system)	For chromium ion levels, no significant differences between four groups. For cobalt ion levels, a significant difference between the groups at 3, 6, 12 and 24 months, with the Zimmer implant showing the highest levels and the Biomet implant the lowest (*p* = 0.027, <0.001, 0.007 and 0.001 at 3, 6, 12 and 24 months, respectively)	Not evaluated
Lardanchet et al. [37]	Comparison of metal ion levels in three different large-head MOM THA systems (Biomet modular M2a-Magnum system, Zimmer Durom LDH system and Wright Conserve Total system)	Cobalt ion levels significantly higher with Conserve Total than with Durom and M2a Magnum (no significant difference between the last two). Chromium ion levels significantly lower with Durom than with Conserve Total. No significant differences for chromium levels between Durom and M2a Magnum or between Conserve Total and M2a Magnum	Better outcome scores in M2a Magnum as compared to Conserve Total (*p* = 0.008, Wilcoxon test) with no significant difference between Durom and M2a Magnum (*p* = 0.22) or between Durom and Conserve Total (*p* = 0.11)
Current study	Comparison of metal ion levels in three different large-head MOM THA systems (Two monoblock systems, Zimmer Durom LDH system and Smith and Nephew Birmingham socket system; one modular system, Depuy Pinnacle socket system)	Metal ions significantly higher in the two monoblock head systems (Zimmer Durom LDH system, and Smith and Nephew Birmingham socket system) as compared to the modular Pinnacle socket system from Depuy	No significant difference in all three systems

Several authors have looked at the effect of increasing head diameter on the wear rates of a MOM THA [46-49]. Unlike MOP THA, the wear rate for MOM THA has been shown to be lower with increasing head diameter. Liu et al. explained that this is based on the fact that a larger diameter head increases the entrainment velocity, thereby improving the lubrication and decreasing wear with a MOM bearing [50]. Bowsher et al. [51] in their study showed a reduction in wear with a size 40 mm bearing when compared to size 28 mm. However, the authors actually found an increase in wear with 56 mm bearings. This may be related to the clearance of the couple. In a study by Leslie et al. [52], there was actually a reduction in wear rates of larger head MOM couples (55 mm head) as compared to smaller heads (39 mm) with similar radial clearances. The authors explained their results based on the reduction in inlet pressure gradients with increasing head size. Thus, multiple factors and not just the head size may affect the wear rate (and hence the metal ion levels) in a MOM THA.

Increasing the femoral head size in THA is beneficial in many respects, especially with regard to joint stability and avoidance of component impingement [20]. In a comprehensive review of the literature published by Cross et al. [53], the authors concluded that increasing femoral head size decreases the risk of postoperative dislocation and improves impingement-free ROM. However, volumetric wear increases with large femoral heads on polyethylene and increases corrosion of the stem in large metal-on-metal modular THA. Also, the authors found that the range of motion and impingement is not much different once the head size goes beyond 36 mm. This is explained by the fact that it is the component impingement that restricts the ROM in small diameter heads, while in the larger diameter heads, it is actually the bony impingement (and not component impingement) that restricts ROM beyond one stage [53]. Thus, there may not be any mechanical advantage (as far as ROM/impingement is concerned) once the head size goes beyond 36 or 40 mm.

With increasing concerns regarding the higher failure rate of large-head MOM THAs, it is important to identify the safe upper limit for femoral head size in MOM THA. Few reports have compared the outcomes of different sized bearings in MOM THR. However, none to our knowledge has actually compared large head (36 to 44 mm) sizes to even larger (>48 mm) sizes as in our study. Pattyn et al. [36] have published a prospective study on comparing three metal-on-metal bearing surfaces, namely the Durom socket, the Birmingham socket and the Metasul total hip system, which is a modular total hip system. This study identified ion levels to be lower in one of the larger head resurfacing groups which appears to conflict with the data of the current study. Bernstein et al. [34] compared two groups of patients, one with head sizes of 28 and 36 mm and another group comparing size 40 and 44 mm heads and found no significant relationship between metal ion levels in whole blood and the size of the femoral heads. They used a similar design to the current study, but the maximum head size examined was only 44 mm, which may be the reason for their conclusion.

Although the short functional outcome scores (WOMAC, SF-36 and Harris Hip Score) were similar in all three MOM THA systems in the current study, metal ions were found to be higher in the larger monoblock heads as compared to the smaller head modular Pinnacle system. One possible explanation for the difference in the blood metal ions could be a mismatch between the head and neck size of the monoblock head implants. A higher mismatch in the head and neck size can lead to increased rotational movement and torque at the head-neck junction [20]. This can, in turn, lead to higher wear at the head neck-junction and higher release of metal ion levels. In fact, some studies have shown increased corrosion and fretting as a result of wear at the trunnion-head interface (thereby increasing metal ion release in the body) as a result of large diameter MOM THA [54,55]. However the concept of increased torque at the head-neck junction with a larger head is just a theoretical explanation and our study can in no way prove this concept.

In the current study, age, BMI, medical comorbidities (smoking, diabetes, etc.) and postsurgery activity levels were not found to have an effect on blood metal ion levels. These results were very much similar to those reported previously [2,20-22,24-37]. Further, the blood metal ion levels were not found to have a statistically significant relationship to anteversion or abduction angle of the acetabular component as measured on the radiographs. This was clearly in contrast to the findings of previous studies that have found higher blood metal ion levels with decreased anteversion and increased abduction angles of the acetabular component [25-28]. One of the potential reasons for this was the relatively smaller number of patients and lack of sufficient power in our study to evaluate this effect.

There were a few limitations of the current study. The cross-sectional cohort design makes it far less ideal as compared to a randomized control trial. Also, the number of patients enrolled in the study was small. This was an exploratory study. A larger study population would have increased the power of our study and enabled us to better evaluate the effect of other factors (like component position) on the level of metal ions in the blood. Our strict exclusion criteria and patients inability to participate in this call back study further reduced the numbers. Another potential limitation is the inability to rule out the effect of different manufacturing techniques utilized by the three companies as partly responsible for the differences in the metal ions. Although we strongly believe that a larger head was the major reason behind higher metal ion levels in the Durom/Birmingham systems, we cannot really conclude that there are no other factors contributing to this difference in the metal ion levels in the three MOM hip systems included in our study. Also, although there was no dislocation in any of the patients in all the three groups, the study is underpowered to make any analysis on this given the small risk of dislocation following total hip arthroplasty now.

In the current series of patients, only one patient underwent a revision procedure. This was following haematogenous infection after a dental abscess which happened a couple of months after our review for the current study. The lead author (JP) actually has a series of 41 Durom MOM THR. Eight of these patients have now undergone revision procedures for implant failure related to prosthetic design. These patients presented with persistent pain and the absence of bony ingrowth in the acetabular socket. None of them had evidence of metallosis at the time of revision. Therefore, whilst in our study the short-term functional results are very similar between the monoblock systems, this may not represent the true overall long-term outcomes. Despite these limitations, this study indicates that there are significant differences in metal ion levels among large head devices, and it provides valuable information that can be used for designing more comprehensive evaluations of factors related to metal ion levels.

## Conclusion

In summary, the current study is one of a few of its kind to compare blood metal ions and short-term clinical outcomes in large-head (36 to 44 mm) modular MOM THA systems with even larger (>48 mm) monoblock MOM THA systems. We have shown higher blood metal ion levels in the larger monoblock MOM THA systems. The Pinnacle type modular systems (with head size in range of 36 to 44 mm) seem to be large enough (similar to ≥48 mm heads) to provide advantages of joint stability, better function and lesser impingement (as against small diameter heads ≤32 mm heads). At the same time, the modular systems have a femoral head that is small enough so as not to cause an excessive increase in metal ion levels in the blood as seen with monoblock head designs with larger metal heads (>48 mm). We believe that when considering a metal-on-metal THR, the use of a monoblock large-head system offers no apparent advantage to the smaller head modular system. In our practice, we are no longer using this design and the safest strategy, when considering MOM bearings, is to use a modular system with a smaller head.

## Competing interests

D. Marshall received a grant from Alberta Health and Wellness/Adhoc consulting in health economics and outcomes research for Optum Insight. B. Burkart is a consultant at Stryker Canada Ltd. D. Kinniburgh received a grant from Alberta Health. The remaining authors declare that they have no competing interests.

## Authors’ contributions

JS and DL participated in the patient review, data analysis and manuscript preparation. KB participated in the data analysis and manuscript preparation and review. PR participated in the study design, patient review, data collection and manuscript preparation. DK participated in the study design, metal ion monitoring and review. PF participated in the study design, statistical analysis and manuscript review. DM participated in the study design and manuscript preparation. BB participated in the data review and manuscript preparation. JP participated in the study conception, study design, data review, manuscript preparation and review. All authors read and approved the final manuscript.

## References

[B1] Pivec R Johnson AJ Mears SC Mont MA Hip arthroplasty Lancet 2012 380 9855 1768 1777 10.1016/S0140-6736(12)60607-2 23021846

[B2] Jacobs JJ Roebuck KA Archibeck M Hallab NJ Glant TT Osteolysis: basic science Clin Orthop Relat Res 2001 393 71 77 11764373 10.1097/00003086-200112000-00008

[B3] Chan FW Bobyn JD Medley JB Krygier JJ Tanzer M The Otto Aufranc Award. Wear and lubrication of metal-on-metal hip implants Clin Orthop Relat Res 1999 369 10 24 10611857 10.1097/00003086-199912000-00003

[B4] Peters CL McPherson E Jackson JD Erickson JA Reduction in early dislocation rate with large-diameter femoral heads in primary total hip arthroplasty J Arthroplasty 2007 22 6 Suppl 2 140 144 17823033 10.1016/j.arth.2007.04.019

[B5] Anissian HL Stark A Gustafson A Good V Clarke IC Metal-on-metal bearing in hip prosthesis generates 100-fold less wear debris than metal-on-polyethylene Acta Orthop Scand 1999 70 6 578 582 10.3109/17453679908997845 10665722

[B6] Doorn PF Campbell PA Worrall J Benya PD McKellop HA Amstutz HC Metal wear particle characterization from metal on metal total hip replacements: transmission electron microscopy study of periprosthetic tissues and isolated particles J Biomed Mater Res 1998 42 1 103 111 10.1002/(SICI)1097-4636(199810)42:1<103::AID-JBM13>3.0.CO;2-M 9740012

[B7] Brodner W Bitzan P Meisinger V Kaider A Gottsauner-Wolf F Kotz R Serum cobalt levels after metal-on-metal total hip arthroplasty J Bone Joint Surg Am 2003 85 11 2168 2173 14630848 10.2106/00004623-200311000-00017

[B8] Rasquinha VJ Ranawat CS Weiskopf J Rodriguez JA Skipor AK Jacobs JJ Serum metal levels and bearing surfaces in total hip arthroplasty J Arthroplasty 2006 21 6 Suppl 2 47 52 16950061 10.1016/j.arth.2006.05.005

[B9] Qu X Huang X Dai K Metal-on-metal or metal-on-polyethylene for total hip arthroplasty: a meta-analysis of prospective randomized studies Arch Orthop Trauma Surg 2011 131 11 1573 1583 10.1007/s00402-011-1325-2 21643799

[B10] Cobb AG Schmalzreid TP The clinical significance of metal ion release from cobalt-chromium metal-on-metal hip joint arthroplasty Proc Inst Mech Eng H 2006 220 2 385 398 10.1243/09544119JEIM78 16669404

[B11] Case CP Langkamer VG Lock RJ Perry MJ Palmer MR Kemp AJ Changes in the proportions of peripheral blood lymphocytes in patients with worn implants J Bone Joint Surg Br 2000 82 5 748 754 10.1302/0301-620X.82B5.9946 10963179

[B12] Davies AP Sood A Lewis AC Newson R Learmonth ID Case CP Metal-specific differences in levels of DNA damage caused by synovial fluid recovered at revision arthroplasty J Bone Joint Surg Br 2005 87 10 1439 1444 16189324 10.1302/0301-620X.87B10.16541

[B13] Hart AJ Skinner JA Winship P Faria N Kulinskaya E Webster D Muirhead-Allwood S Aldam CH Anwar H Powell JJ Circulating levels of cobalt and chromium from metal-on-metal hip replacement are associated with CD8+ T-cell lymphopenia J Bone Joint Surg Br 2009 91 6 835 842 19483243 10.1302/0301-620X.91B6.21844

[B14] Dayan AD Paine AJ Mechanisms of chromium toxicity, carcinogenicity and allergenicity: review of the literature from 1985 to 2000 Hum Exp Toxicol 2001 20 9 439 451 10.1191/096032701682693062 11776406

[B15] Keegan GM Learmonth ID Case CP Orthopaedic metals and their potential toxicity in the arthroplasty patient: a review of current knowledge and future strategies J Bone Joint Surg Br 2007 89 5 567 573 17540737 10.1302/0301-620X.89B5.18903

[B16] Tower SS Arthroprosthetic cobaltism: neurological and cardiac manifestations in two patients with metal-on-metal arthroplasty: a case report J Bone Joint Surg Am 2010 92 17 2847 2851 21037026 10.2106/JBJS.J.00125

[B17] Tower SS Arthroprosthetic cobaltism associated with metal on metal hip implants BMJ 2012 344 e430 10.1136/bmj.e430 22252702

[B18] Machado C Appelbe A Wood R Arthroprosthetic cobaltism and cardiomyopathy Heart Lung Circ 2012 21 11 759 760 10.1016/j.hlc.2012.03.013 22520206

[B19] Jacobs JJ Urban RM Gilbert JL Skipor AK Black J Jasty M Galante JO Local and distant products from modularity Clin Orthop Relat Res 1995 319 94 105 7554654

[B20] Lavigne M Belzile EL Roy A Morin F Amzica T Vendittoli PA Comparison of whole-blood metal ion levels in four types of metal-on-metal large-diameter femoral head total hip arthroplasty: the potential influence of the adapter sleeve J Bone Joint Surg Am 2011 93 Suppl 2 128 136 21543702 10.2106/JBJS.J.01885

[B21] Gill IP Webb J Sloan K Beaver RJ Corrosion at the neck-stem junction as a cause of metal ion release and pseudotumour formation J Bone Joint Surg Br 2012 94 7 895 900 22733942 10.1302/0301-620X.94B7.29122

[B22] Meyer H Mueller T Goldau G Chamaon K Ruetschi M Lohmann CH Corrosion at the cone/taper interface leads to failure of large-diameter metal-on-metal total hip arthroplasties Clin Orthop Relat Res 2012 470 11 3101 3108 10.1007/s11999-012-2502-5 22864616 PMC3462871

[B23] Garbuz DS Tanzer M Greidanus NV Masri BA Duncan CP The John Charnley Award: metal-on-metal hip resurfacing versus large-diameter head metal-on-metal total hip arthroplasty: a randomized clinical trial Clin Orthop Relat Res 2010 468 2 318 325 10.1007/s11999-009-1029-x 19697090 PMC2806981

[B24] Brodner W Grübl A Jankovsky R Meisinger V Lehr S Gottsauner-Wolf F Cup inclination and serum concentration of cobalt and chromium after metal-on-metal total hip arthroplasty J Arthroplasty 2004 19 8 Suppl 3 66 70 15578556 10.1016/j.arth.2004.09.003

[B25] De Haan R Pattyn C Gill HS Murray DW Campbell PA De Smet K Correlation between inclination of the acetabular component and metal ion levels in metal-on-metal hip resurfacing replacement J Bone Joint Surg Br 2008 90 10 1291 1297 18827237 10.1302/0301-620X.90B10.20533

[B26] Hart AJ Buddhdev P Winship P Faria N Powell JJ Skinner JA Cup inclination angle of greater than 50 degrees increases whole blood concentrations of cobalt and chromium ions after metal-on-metal hip resurfacing Hip Int 2008 18 3 212 219 18924077 10.1177/112070000801800304

[B27] Hart AJ Skinner JA Henckel J Sampson B Gordon F Insufficient acetabular version increases blood metal ion levels after metal-on-metal hip resurfacing Clin Orthop Relat Res 2011 469 9 2590 2597 10.1007/s11999-011-1930-y 21656317 PMC3148360

[B28] Langton DJ Jameson SS Joyce TJ Webb J Nargol AV The effect of component size and orientation on the concentrations of metal ions after resurfacing arthroplasty of the hip J Bone Joint Surg Br 2008 90 9 1143 1151 18757952 10.1302/0301-620X.90B9.20785

[B29] Clarke MT Lee PT Arora A Villar RN Levels of metal ions after small-and large-diameter metal-on-metal hip arthroplasty J Bone Joint Surg Br 2003 85 6 913 917 12931818

[B30] Daniel J Ziaee H Salama A Pradhan C McMinn DJ The effect of the diameter of metal-on-metal bearings on systemic exposure to cobalt and chromium J Bone Joint Surg Br 2006 88 4 443 448 16567776 10.1302/0301-620X.88B4.17355

[B31] Daniel J Ziaee H Pradhan C McMinn DJ Systemic metal exposure in large-and small-diameter metal-on-metal total hip replacements Orthopedics 2008 31 12 Suppl 2 379 390 19298017

[B32] Antoniou J Zukor DJ Mwale F Minarik W Petit A Huk OL Metal ion levels in the blood of patients after hip resurfacing: a comparison between twenty-eight and thirty-six-millimeter-head metal-on-metal prostheses J Bone Joint Surg Am 2008 90 Suppl 3 142 148 18676949 10.2106/JBJS.H.00442

[B33] Vendittoli PA Roy A Mottard S Girard J Lusignan D Lavigne M Metal ion release from bearing wear and corrosion with 28 mm and large-diameter metal-on-metal bearing articulations: a follow-up study J Bone Joint Surg Br 2010 92 1 12 19 20044673 10.1302/0301-620X.92B1.22226

[B34] Bernstein M Walsh A Petit A Zukor DJ Huk OL Antoniou J Femoral head size does not affect ion values in metal-on-metal total hips Clin Orthop Relat Res 2011 469 6 1642 1650 10.1007/s11999-010-1630-z 20963530 PMC3094634

[B35] Hallows RK Pelt CE Erickson JA Peters CL Serum metal ion concentration: comparison between small and large head metal-on-metal total hip arthroplasty J Arthroplasty 2011 26 8 1176 1181 10.1016/j.arth.2010.11.004 21236627

[B36] Pattyn CA Lauwagie SN Verdonk RC Whole blood metal ion concentrations in correlation with activity level in three different metal-on-metal bearings J Arthroplasty 2011 26 1 58 64 10.1016/j.arth.2009.11.007 20171052

[B37] Lardanchet JF Taviaux J Arnalsteen D Gabrion A Mertl P One-year prospective comparative study of three large-diameter metal-on-metal total hip prostheses: serum metal ion levels and clinical outcomes Orthop Traumatol Surg Res 2012 98 3 265 274 10.1016/j.otsr.2011.11.009 22480865

[B38] Pei KL Kinniburgh DW Butlin L Faris P Lee D Marshall DA Oliver MC Parker R Powell JN Railton P Smith J An ORS-ICP-MS method for monitoring trace levels of cobalt and chromium in whole blood samples from hip arthroplasty patients with metal-on-metal prostheses Clin Biochem 2012 45 10–11 806 810 22484458 10.1016/j.clinbiochem.2012.03.025

[B39] Back DL Young DA Shimmin AJ How do serum cobalt and chromium levels change after metal-on-metal hip resurfacing? Clin Orthop Relat Res 2005 438 177 181 16131888 10.1097/01.blo.0000166901.84323.5d

[B40] MacDonald SJ Can a safe level for metal ions in patients with metal-on-metal total hip arthroplasties be determined? J Arthroplasty 2004 19 8 Suppl 3 71 77 15578557 10.1016/j.arth.2004.09.008

[B41] Alimonti A Bocca B Mannella E Petrucci F Zennaro F Cotichini R D’Ippolito C Agresti A Caimi S Forte G Assessment of reference values for selected elements in a healthy urban population Ann Ist Super Sanita 2005 41 2 181 187 16244391

[B42] De Smet K De Haan R Calistri A Campbell PA Ebramzadeh E Pattyn C Gill HS Metal ion measurement as a diagnostic tool to identify problems with metal-on-metal hip resurfacing J Bone Joint Surg Am 2008 90 Suppl 4 202 208 18984732 10.2106/JBJS.H.00672

[B43] Moroni A Savarino L Cadossi M Baldini N Giannini S Does ion release differ between hip resurfacing and metal-on-metal THA? Clin Orthop Relat Res 2008 466 3 700 707 10.1007/s11999-007-0106-2 18196364 PMC2505207

[B44] Updated Guidance on Large Diameter Metal on Metal bearing Total Hip Replacements British Orthopaedic Association advice to members 2011 Available at: http://www.britishhipsociety.com/pdfs/BHS_MOM_THR.pdf. Accessed on January 26, 2013

[B45] Malviya A Ramaskandhan J Holland JP Lingard EA Metal-on-metal total hip arthroplasty J Bone Joint Surg Am 2010 92 7 1675 1683 10.2106/JBJS.I.01426 20595577

[B46] Smith SL Dowson D Goldsmith AA The effect of femoral head diameter upon lubrication and wear of metal-on-metal total hip replacements Proc Inst Mech Eng H 2001 215 2 161 170 11382075 10.1243/0954411011533724

[B47] Hu XQ Isaac GH Fisher J Changes in the contact area during the bedding-in wear of different sizes of metal on metal hip prostheses Biomed Mater Eng 2004 14 2 145 149 15156105

[B48] Dowson D Hardaker C Flett M Isaac GH A hip joint simulator study of the performance of metal-on-metal joints: part II: design J Arthroplasty 2004 19 8 Suppl 3 124 130 15578566 10.1016/j.arth.2004.09.016

[B49] Affatato S Leardini W Jedenmalm A Ruggeri O Toni A Larger diameter bearings reduce wear in metal-on-metal hip implants Clin Orthop Relat Res 2007 456 153 158 17065844 10.1097/01.blo.0000246561.73338.68

[B50] Bowsher JG Hussain A Williams PA Shelton JC Large head diameters have the potential to reduce ion release in metal-on metal hip wear simulations ORS 2005 Washington DC

[B51] Liu F Jin Z Roberts P Grigoris P Importance of head diameter, clearance and cup wall thickness in elastohyrodynamic lubrication analysis of metal-on-metal hip resurfacing prostheses J Eng Med 2006 220 695 704 10.1243/09544119JEIM172 16961189

[B52] Leslie I Williams S Brown C Isaac G Jin Z Ingham E Fisher J Effect of bearing size on the long-term wear, wear debris, and ion levels of large diameter metal-on-metal hip replacements-an in vitro study J Biomed Mater Res B Appl Biomater 2008 87 1 163 172 18386846 10.1002/jbm.b.31087

[B53] Cross MB Nam D Mayman DJ Ideal femoral head size in total hip arthroplasty balances stability and volumetric wear HSS J 2012 8 3 270 274 10.1007/s11420-012-9287-7 24082871 PMC3470670

[B54] Bolland BJ Culliford DJ Langton DJ Millington JP Arden NK Latham JM High failure rates with a large-diameter hybrid metal-on-metal total hip replacement: clinical, radiological and retrieval analysis J Bone Joint Surg Br 2011 93 5 608 615 21511925 10.1302/0301-620X.93B5.26309

[B55] Dyrkacz RMR Turgeon T Ojo O Brandt JM Wyss U Head size affects corrosion behavior in artificial hip joints 2012 Transactions of the 2012 Orthopaedic Research Society

